# Tactile Perception Object Recognition Based on an Improved Support Vector Machine

**DOI:** 10.3390/mi13091538

**Published:** 2022-09-17

**Authors:** Xingxing Zhang, Shaobo Li, Jing Yang, Yang Wang, Zichen Huang, Jinhu Zhang

**Affiliations:** State Key Laboratory of Public Big Data, Guizhou University, Guiyang 550025, China

**Keywords:** tactile perception, object recognition, SVM, machine learning algorithms

## Abstract

Tactile perception is an irreplaceable source of information for humans to explore the surrounding environment and has advantages over sight and hearing in processing the material properties and detailed shapes of objects. However, with the increasing uncertainty and complexity of tactile perception features, it is often difficult to collect highly available pure tactile datasets for research in the field of tactile perception. Here, we have proposed a method for object recognition on a purely tactile dataset and provide the original tactile dataset. First, we improved the differential evolution (DE) algorithm and then used the DE algorithm to optimize the important parameter of the Gaussian kernel function of the support vector machine (SVM) to improve the accuracy of pure tactile target recognition. The experimental comparison results show that our method has a better target recognition effect than the classical machine learning algorithm. We hope to further improve the generalizability of this method and provide an important reference for research in the field of tactile perception and recognition.

## 1. Introduction

Tactile perception is an important part of the autonomous operation of a robot’s dexterous hand. It provides information on the force and surface characteristics of a robot’s finger and the target contact point [1,2]. Giving robots the ability to accurately recognize objects with tactile perception is an urgent scientific challenge in the field of intelligent robot research [3]. Machine learning algorithms have powerful data training capabilities and can better complete the classification of different data [4]. Classic machine learning algorithms (such as the k-neighbor algorithm (KNN) [5,6], decision tree (DT) [7], Gauss Bayes (GNB) [8], support vector machine (SVM) [9,10], random forest (RF) [11], etc.) can be applied to the object recognition problem of tactile sensing data. The latest advances in robotic tactile sensing have promoted the development of many scientific computing technologies. The application of these technologies to the important sensory channel of touch has important practical significance for the leap forward in the development of intelligent robots.

In recent years, tactile sensor technology has developed rapidly, and there have been many advances in performance and applications [12,13,14]. Tactile sensor technology can detect the force of the target in real time and apply the detected tactile pressure data to the object recognition problem [15]. Alin Drimusa, Gert Kootstrab, et al. [16] demonstrated the application of tactile sensors in active target recognition systems. This research was based on the k-nearest neighbor classifier, which uses dynamic time warp to calculate the distance between different time series, which can successfully identify the target. Subramanian Sundaram et al. [17] designed a retractable tactile glove with 548 sensors on the entire hand. They built a deep convolutional neural network model to process and analyze the tactile data map. Finally, the classification of 26 types of objects was realized.

Recent studies have shown that tactile sensing target recognition problems can also use the spatio-temporal hierarchical matching pursuit (ST-HMP) unsupervised feature learning method [18], deep convolutional neural network model method [19], and efficient codebook formula clustering method (LDS-FCM) [20] to solve them. Brayan S. Zapata-Impata [21] and others used a multi-array tactile sensor grasping experiment platform to carry out target grasping experiments to obtain tactile data and identify targets. The platform was obtained by installing three BioTac SP tactile sensors developed by SynTouch on the top of the index finger, middle finger, and thumb of the shadow dexterous manipulator developed by Shadow Robot. Satoshi Funabashi et al. [22] studied the tactile target recognition problem of relatively densely distributed force vector measurements. First, the UsKin tactile sensor was embedded in Alelgo’s hand. A total of 240 three-axis force vector measurements were provided in all fingers to obtain time series training and test data. Then, object recognition was realized by simple feedforward, recursive, and convolutional neural networks to identify targets. The recognition rate of 20 targets can be as high as 95%. Chunfang Liu et al. [20] proposed a new efficient codebook formula clustering method (LDS-FCM) for spatio-temporal tactile target recognition. The VLAD method was used to obtain the final feature description of the tactile data, and the experiment was verified through five public databases.

The above methods, although validated on different datasets, used processed public datasets. These datasets are insufficient to guarantee that they are equally effective at solving more primitive and diverse recognition problems. If it is experimentally verified in the dataset collected by oneself, it can better demonstrate the generalization ability of the above method.

In response to the above problems, we have provided the original tactile dataset for target recognition and proposed a method to optimize the support vector machine. We have also verified the effectiveness of the method through experiments. We have achieved target recognition through data collection, data processing, data analysis, and other processes.

The main contributions of this paper lie in the following aspects:

(1) We have realized the effective application of different machine learning algorithms in the target recognition of pure tactile perception. We have proposed an improved support vector machine algorithm for tactile perception recognition and verified the effectiveness of the method.

(2) We used grips instead of single touch to record tactile pressure data, as grip is more useful than touch for the operation of intelligent robots and external factors have very little effect on such an experiment.

(3) A new, real, pure tactile grasping perception dataset is released in this study, which provides a useful data resource for researchers in related fields to further study the methods of learning tactile perception.

The rest of this paper is organized as follows: Section 2 introduces the classic machine learning algorithm and proposes an improved SVM algorithm. Section 3 outlines the designs and creation of the tactile dexterous-hand grasping platform for collecting tactile perception data. Section 4 explains the design and production of the tactile dataset and compares and analyzes the experimental results. The final section summarizes the research content.

## 2. An SVM Optimization Method Based on Tactile Perception Target Recognition

The differential evolution algorithm is simple in structure and easy to execute. It has the advantages of high optimization efficiency, simple parameter setting, and good robustness [23,24]. Therefore, we selected the DE algorithm in the intelligent optimization algorithm to optimize the machine learning algorithm. We proposed a method to optimize the SVM using a modified DE algorithm. This method is used to solve the object recognition problem. We optimized the parameter γ of the Gaussian kernel function of the SVM (see Equation (9) for the calculation of the Gaussian kernel function) in order to improve the training accuracy of the SVM classification model.

### 2.1. Improved Differential Evolution Algorithm

#### 2.1.1. The Original Differential Evolution Algorithm

(1) Generate an initial population. Initial population $\left\{x_{i}(0)|x_{j,i}^{L}\leq x_{j,i}(0)\leq x_{j,i}^{U},i=1,2,\cdots ,NP;j=1,2,\cdots ,D\right\}$ Randomly generated:

(1) $$x_{j,i}(0)=x_{j,i}^{L}+rand(0,1)\cdot (x_{j,i}^{U}-x_{j,i}^{L})$$

where $x_{i}(0)$ represents the *i-th* individual of the 0-*th* generation in the population, and $x_{j,i}(0)$ represents the *j-th* “gene” of the *i-th* individual in the 0-*th* generation. NP represents the population size, and rand(0,1) represents a random number uniformly distributed in the interval (0, 1).

(2) Mutations. DE achieves individual variation through a differential strategy. Choosing the differential strategy shown in (2).

(2) $$v_{i}(g+1)=x_{r1}(g)+F(x_{r2}(g)-x_{r3}(g))$$

where $i\neq r_{1}\neq r_{2}\neq r_{3}$, *F* is the crossover factor, and the value is generally optional (0.2~1.5). $x_{1}(g)$ represents the *i-th* individual in the *g-th* generation population.

(3) Cross. Perform inter-individual crossover operations on the *g-th* population $\left\{x_{i}(g)\right\}$ and its mutant intermediate $\left\{v_{i}(g+1)\right\}$

(3) $$u_{j,i}(g+1)=\{\begin{array}{c} v_{j,i}(g+1),ifrand(0,1)\leq CRorj=j_{rand}\\ x_{j.i}(g+1),otherwise \end{array}$$

where *CR* is the crossover probability, and the value is generally (0~1), and $j_{rand}$ is a random integer of [1,2,⋯,D].

(4) Choose. DE uses a greedy algorithm to select individuals who enter the next-generation population:

(4) $$x_{i}(g+1)=\{\begin{array}{c} u_{i}(g+1),iff(u_{i}(g+1))\leq f(x_{i}(g))\\ x_{i}(g),otherwise \end{array}$$

#### 2.1.2. Improved Differential Evolution Algorithm (DE)

In the optimization process, the effects that the DE algorithm needs to achieve are different in the initial, mid, and late stages. If the mutation factor *F* is set to a constant, the algorithm can easily fall into the local optimum, reducing the convergence speed and search accuracy. Therefore, we adjusted *F* linearly, as shown in Formula (5). *F* will change as the number of iterations increases.

(5) $$F=(F_{1}-F_{2})\frac{G-g}{G}+F_{2}$$

where *F*_1_ and *F*_2_ are the maximum and minimum mutation factors, *G* is the maximum number of iterations, and *g* is the current number of iterations.

As the cross factor *CR* has a greater impact on selecting the next-generation population individuals, a constant *CR* is difficult to improve the optimization efficiency of the DE algorithm. Therefore, we adaptively adjusted *CR* according to Formula (6).

(6) $$CR=CR_{2}+\frac{CR_{1}-CR_{2}}{G}g$$

Here, *CR*_1_ and *CR*_2_ are the maximum and minimum crossover factors, respectively. *G* is the maximum number of iterations. *g* is the current number of iterations.

### 2.2. Improved Differential Evolution Algorithm (DE) to Optimize Support Vector Machine (SVM)

#### 2.2.1. The Original Support Vector Machine (SVM)

The SVM model represents training targets as points in space, which were mapped to a picture, separated by a clear, widest possible interval to distinguish two categories [25]. Subsequently, the new targets were mapped into the same space. The categories they belonged to were predicted based on which side of the interval they fell on.

The SVM found a separating hyperplane in the distributed data as the decision boundary so that the classification error of the model on the data was as small as possible [26]. First, the support vector was found, then the distance between the support vector and the separation plane was maximized, and finally, the most suitable hyperplane was found.

The classification decision function is:

(7) $$f\left(x\right)=sign(w^{T}*x+b)$$

The optimization objective function is:

(8) $$\underset{\left(w,b\right)}{\min}\left\|w\right\|\;\;\;\;\;\;\;\;\;\;\;\;s.t.\;\;\;\;y_{i}\left(w^{T}*X_{i}+b\right)\geq \delta ,i=1,\ldots ,m$$

where *w* is the vector perpendicular to the hyperplane. *x* is the support vector. *b* is the intercept.

#### 2.2.2. Improved Differential Evolution Algorithm to Optimize Support Vector Machine (DESVM)

Because the sample feature dimension was not high and the number was not large, we adopted the Gaussian kernel function as the kernel function of the support vector machine. We used an improved DE algorithm to optimize the parameter *γ* of the SVM kernel function so that the recognition effect of the SVM reached the best result.

The Gaussian kernel function formula is as follows:

(9) $$k(x,y)=\exp\left(-\gamma {\left\|x-y\right\|}^{2}\right)$$

Here, *γ* is a nuclear parameter.

**Figure 1:**
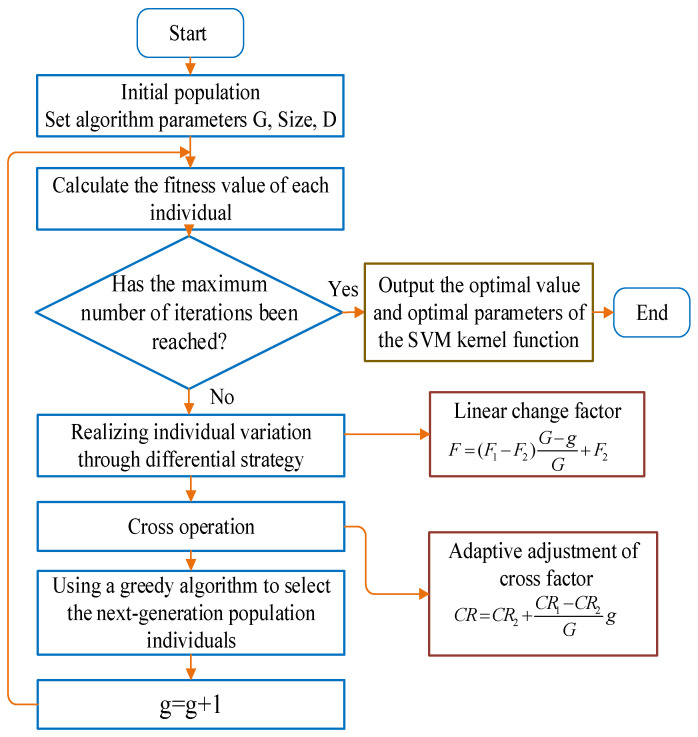
DE algorithm optimization SVM flow chart.

The process of the DE algorithm optimization SVM is shown in Figure 1.

The relevant parameters of the DE algorithm were set as follows: the maximum number of generations, G = 50; the population size, Size = 50; the dimension of the search space, D = 4; the mutation factor, *F;* and the cross factor, *CR* were obtained by linear equations. The purpose of optimizing the parameters of the support vector machine was to maximize the accuracy of classification. Therefore, the fitness value of the DE algorithm was set as: fit = accuracy (classification accuracy). After the optimization of the DE algorithm, the optimal value of the objective function and the optimal parameters of the SVM were finally obtained.

## 3. Tactile Data Collection Platform for the Dexterous Hand

### 3.1. Construction of the Data Collection Platform

**Figure 2:**
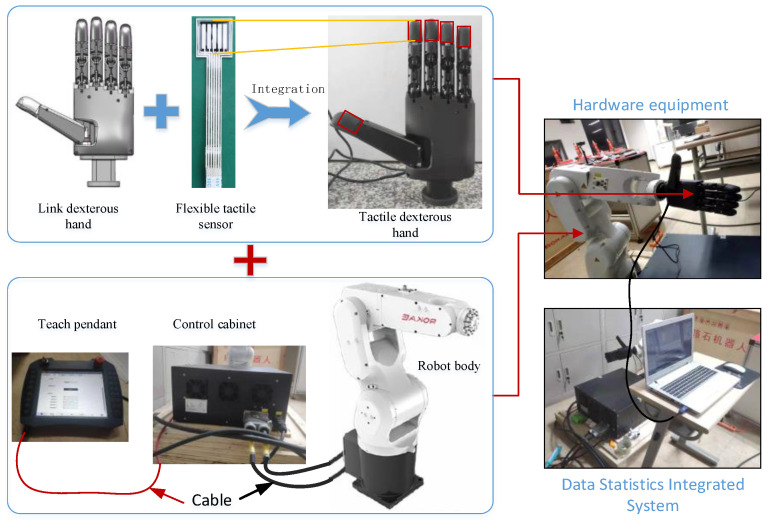
Tactile data collection platform.

Because effective tactile perception data requires high sensitivity and stability of tactile sensors, there are fewer relevant and useful datasets in the field of tactile perception research. In order to collect pure tactile datasets for object recognition, we built a dexterous-hand grasping experiment platform, as shown in Figure 2. The multi-array tactile sensor grasping platform consisted of a robotic arm developed by the RAKAE Robot Company, a linkage-driven dexterous hand developed by the Qingrui Boyuan Company, and five tactile sensors.

The connecting rod dexterous hand adopted a 5-finger design and a connecting rod transmission method, in which the thumb had 2 degrees of freedom, the other fingers had 1 degree of freedom, and the 5 fingers had a total of 6 degrees of freedom to achieve the perfect simulation of a human hand. The fingertip was equipped with a multi-array tactile sensor to sense the grip strength in real time. It also can perform a variety of grasping operations with high precision.

The main materials of the tactile sensors included: a flexible pressure-sensitive material with high sensitivity and a low relaxation time, flexible interface transition materials with high stability, flexible cladding materials with good elasticity and high mechanical strength, and a flexible interconnect material between the nodes. At the same time, combined with CMOS process technology, a composite sensing structure was realized by depositing polymer materials on the substrate of the CMOS tactile sensor readout chip to form a monolithic tactile sensing unit (i.e., node). The flexible technology is used to realize the communication between interconnected sensing units and the signal readout.

First, a polydimethylsiloxane polymer with good elasticity and strong plasticity was used as the base material. These carbon nanotubes were used as the conductive filler of the pressure-sensitive composite material to synthesize a new type of pressure-sensitive composite material with piezoresistive properties. The composite material was prepared into an array flexible tactile sensor with the characteristics of good flexibility, strong fatigue resistance, large dynamic range, and many array units. Then, the tactile sensor, the readout circuit with ultra-wide dynamic range, the sensor interface circuit with stepped resolution, and the main signal processing chip were integrated to form a complete sensing system.

**Figure 3:**
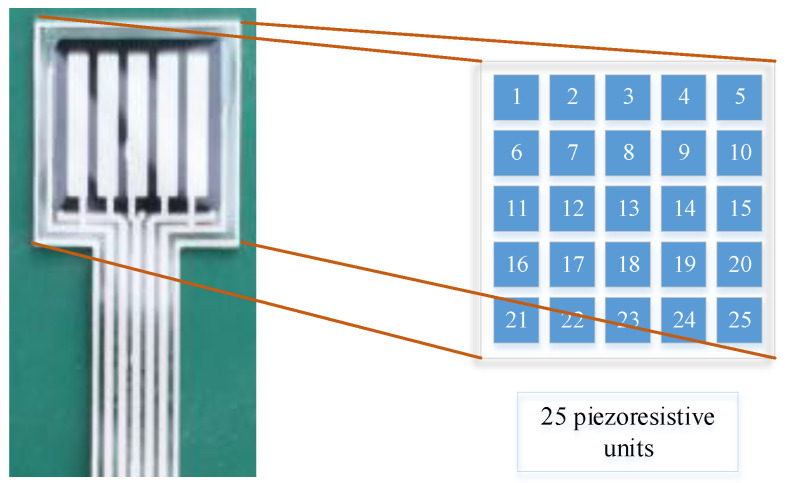
Distribution of each piezoresistive unit of the tactile sensor.

The upper and lower electrodes of the fingertip tactile sensor were designed in the form of “five horizontal and five vertical” so that the whole piece of pressure-sensitive material is automatically divided into a 5 × 5 matrix, that is, 25 piezoresistive units. The schematic diagram of their unit positions and numbers is shown in Figure 3.

The linkage-driven dexterous hand, as a human robot or manipulator end-manipulation tool, can adaptively grasp objects of complex shapes and operate complex tasks in the special environment of industrial production.

This dexterous hand is able to perform a variety of grasping operations with high precision owing to a multi-array pressure sensor at the tip of the fingertip. Linked ambidextrous hands are powered by 8.4 V DC and are suitable for mobile applications.

The ROKAE robot can ensure flexible movement in six degrees of freedom in space. It performs with high precision and at high speed. Its working range is 1206 mm. The repeated positioning accuracy can reach 0.05 mm, which can ensure the smoothness and robustness of the robot trajectory during the experiment and improve the reliability of the data.

### 3.2. Tactile Data Collection Experiment

**Figure 4:**
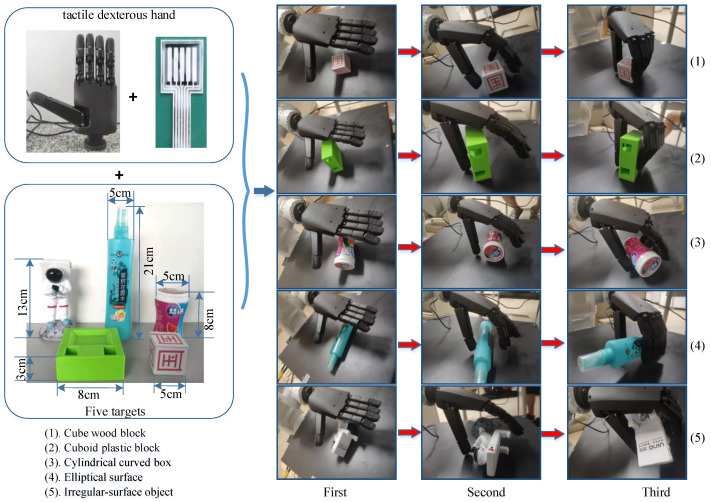
Dataset collection experiment.

We used the teach pendant to control the movement of the ROKAE robot. The ROKAE robot moved to the grasping position with the tactile dexterous hand, and then it performed grasping experiments. The back end of the system recorded the pressure data from the tactile sensor in real time during the grasping process. An example of our grasping experiment is shown in Figure 4. We carried out 100 repeated grasping experiments for each type of object. A total of 500 repeated grasping experiments were carried out. Finally, we obtained the original dataset for tactile perception of five types of objects.

**Figure 5:**
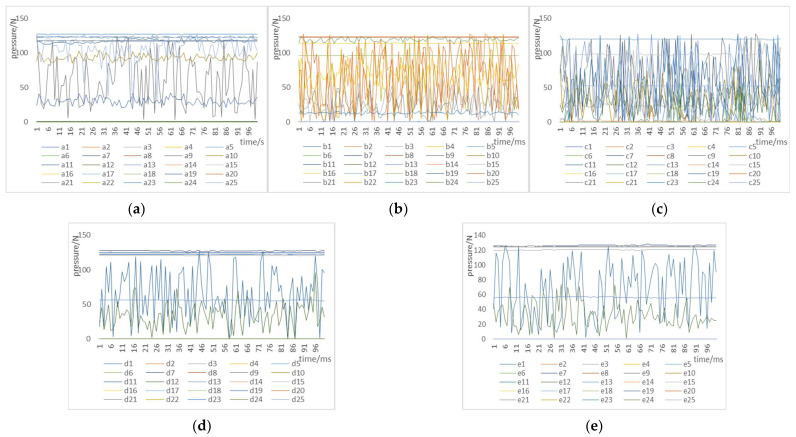
The pressure data curve of five fingers when grabbing an irregular surface object. (**a**) The pressure data map for thumbs. (**b**) The pressure data map of the index finger. (**c**) The pressure data map of the middle finger. (**d**) The pressure data map of the ring finger. (**e**) The pressure data map of the little finger.

Figure 5 shows the variation trend in the pressure data within 100 ms for five fingers of the dexterous hand when grasping the irregular-surface object.

### 3.3. Data Preprocessing

In order to improve the tactile perception data mining analysis, reduce time, reduce cost, and improve quality, we needed to perform data preprocessing on the tactile data collected by tactile sensors.

First, we replaced the mutated data in Figure 5 with the mean value of the tactile data for the fingers within 100 ms and used manual interpolation to preprocess the data. Second, we performed min-max normalization and z-score normalization on the collected tactile perception raw data [27]; the specific methods are as follows:

(1) Min-max normalization

The following calculations were performed for each attribute. Let minA and maxA be the minimum and maximum values of attribute A, respectively. An original value *x* of A was mapped to a value *x*′ in the interval [0, 1] through min-max normalization. The calculation method is as shown (10):

(10) $${x^{\prime }}_{i}=\frac{x_{i}-x_{\min}}{x_{\max}-x_{\min}}$$

(2) The z-score normalization

(11) $$\mu =\frac{1}{N}\sum_{i=1}^{N}x_{i}$$

(12) $$\sigma =\sqrt{\frac{1}{N}\sum_{i=1}^{N}{\left(x_{i}-\mu \right)}^{2}}$$

(13) $${x^{\prime }}_{i}=\frac{x_{i}-\mu }{\sigma }$$

Data standardization was performed based on the mean and standard deviation of the original data. The original value *x* of A was normalized to *x*′ using the z-score. The z-score normalization method is suitable for situations where the maximum and minimum values of attribute A are unknown or when there are outlier data beyond the value range. The calculation method for z-score normalization is shown in (13). *μ* represents the mean. *σ* represents the standard deviation.

## 4. Experimental Results and Analysis

In our experiments, all calculations were performed using a computer with a 64 GB GPU (NVIDIA GeForce RTX 3090) and a Windows 10 operating system. The entire experiment was completed in Anaconda. We used Python language to realize the tactile recognition problem based on machine learning.

### 4.1. Division of the Tactile Perception Dataset

The dataset includes five types of objects. These five types of goals are shown in Figure 4, including (1) a cube wood block, (2) a cuboid plastic block, (3) a cylindrical curved box, (4) an elliptical surface, and (5) an irregular surface object.

We divided the dataset according to the ratio of training samples and test samples to 7:3. Then, we input the training set samples and test set samples for the tactile perception data on five types of objects into KNN, SVM, RF, GNB, DT, and our proposed DESVM, respectively. The five algorithm models were trained and tested. The accuracy of object recognition was finally obtained. The specific division of the dataset is shown in Table 1.

**Figure 6:**
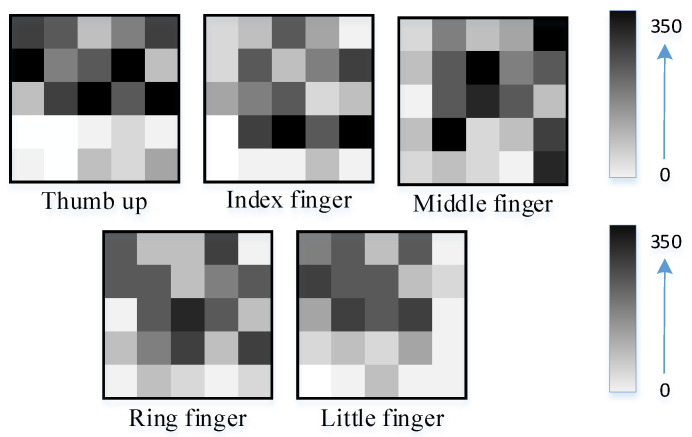
Tactile data map of five fingers.

Figure 6 shows the tactile map of a moment in the grabbing of a cylindrical curved box experiment. The tactile map illustrates the comparison of the amount of pressure data collected by five tactile sensors (a total of 25 × 5 = 125 piezoresistive units). The value 0~350 represents the pressure range of the dexterous hand when grasping the object. The unit is N.

### 4.2. Comprehensive Evaluation Method

Machine learning algorithms are continuously derived and optimized on the basis of traditional algorithms. Various new algorithms have appeared, but the comprehensive performance evaluation indicators of these algorithms are almost the same [28,29]. We use four performance evaluation indicators (i.e., accuracy, precision, recall, and F1) to evaluate the model performance of the classification model.

Accuracy was used to measure the accurate proportion of all samples classified. It can well express the probability of accurate classification. The calculation method for accuracy is shown in Formula (14). The calculation result for precision is shown in Formula (15). Recall is used to measure the probability that a positive sample is correctly predicted among all positive samples. Its calculation method is shown in Formula (16). The F1-score is the harmonic mean of *precision* and *recall*. Its calculation method is shown in Formula (17).

(14) $$Acc=\frac{TP+TN}{TP+FN+FP+TN}$$

(15) $$Precision=\frac{TP}{TP+FP}$$

(16) $$Recall=\frac{TP}{TP+FN}$$

(17) $$F1-score=\frac{2Recall*Precision}{Recall+Precision}$$

Theoretically speaking, when the accuracy rate, *precision* rate, and *recall* rate are close to 1, the prediction effect of the machine learning classification algorithm is better and the prediction is more accurate [27]. However, it is difficult to achieve such an effect in practical applications, especially in the multi-object classification problem for more complex tactile features, where it is almost impossible to achieve. Moreover, the *precision* rate and the *recall* rate often affect each other, one is higher and the other is lower, so in practical applications, an appropriate balance should be made according to specific needs.

### 4.3. Object Recognition Result Analysis

The five machine learning algorithms, KNN, SVM, RF, GNB, and XDESVM, have different effects on classifying five types of objects with different shapes. In order to obtain the best classification effect among the five algorithms, ten groups of experiments were conducted, and the average experimental results are shown in Table 2.

As can be seen from Table 2, the recall and F1 values for GNB are 12.96% and 14.11% lower than for SVM, and 12.86% and 14.45% lower than for RF, respectively. This shows that compared with DT and GNB, the three algorithms KNN, RF, and SVM are more stable. The reason is that GNB and DT assume that the properties are independent of each other, but this assumption is often not true for real haptic data. This has a certain impact on the correct recognition and classification of GNB and DT models. Therefore, the performance of GNB and DT in the object recognition task of tactile perception is not ideal. In addition, the four performance evaluation indicators after training and testing on tactile data samples by SVM and RF are more uniform. All four performance evaluation indicators are better than for KNN. The four performance evaluation indicators, accuracy, precision, recall, and F1, after training with five types of objects by SVM, reached 98.50%, 98.21%, 98.56%, and 98.22%, respectively. With the exception of the F1-score, the other three indicators were a little higher than for RF.

In this paper, the improved DE algorithm was used to optimize the kernel function parameters of SVM, which improves the classification effect of the object.

**Figure 7:**
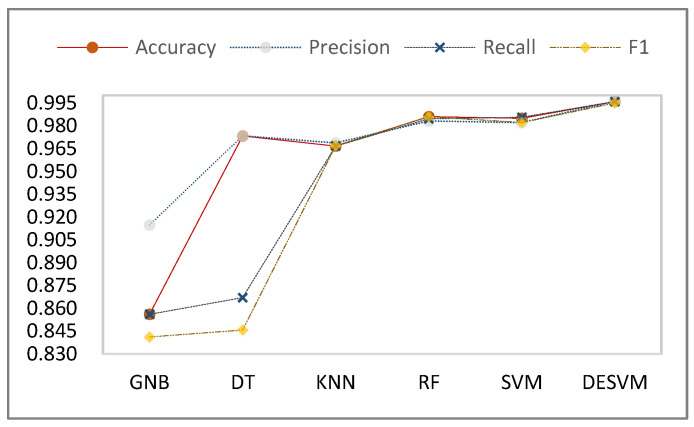
Comparison of experimental results of comprehensive evaluation indicators of six algorithms.

The tactile recognition accuracy for the five types of objects we collected reached 99.60%, the precision reached 99.61%, the recall rate reached 99.60%, and the F1-score reached 99.50%. During training on our collected haptic dataset, DESVM performed better than SVM. The accuracy, precision, recall, and F1-score for object recognition were improved by 1.10%, 1.40%, 1.16%, and 1.32%, respectively. All four indicators improved, which shows that the method proposed in this paper can effectively improve the effect of object classification. The comparison of five machine learning algorithm evaluation index values is shown in Figure 7. The changing trend in the line graph clearly shows the classification effect of the five machine learning algorithms on the five types of objects based on the tactile perception data.

In order to ensure the correctness of the machine learning algorithm in the application of tactile perception recognition, ten-fold hierarchical cross-validation was used to test and evaluate the accuracy of these six classification algorithm models. The experimental results are shown in Table 3.

**Figure 8:**
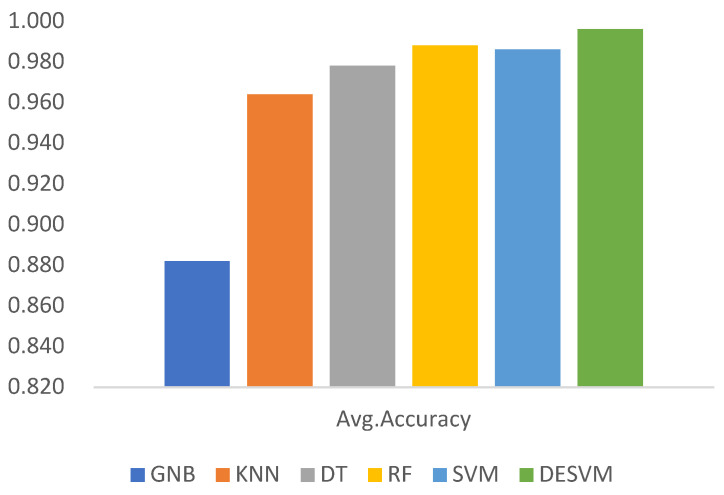
Comparison of algorithm’s accuracy evaluation by ten-fold hierarchical cross-validation.

As shown in Figure 8, the evaluation results show that the improved support vector machine had the highest accuracy for tactile perception object recognition, reaching 99.60%, while the evaluation results of other machine learning algorithms were not very satisfactory, indicating that the optimized support vector machine’s use for tactile perception research has practical significance.

**Figure 9:**
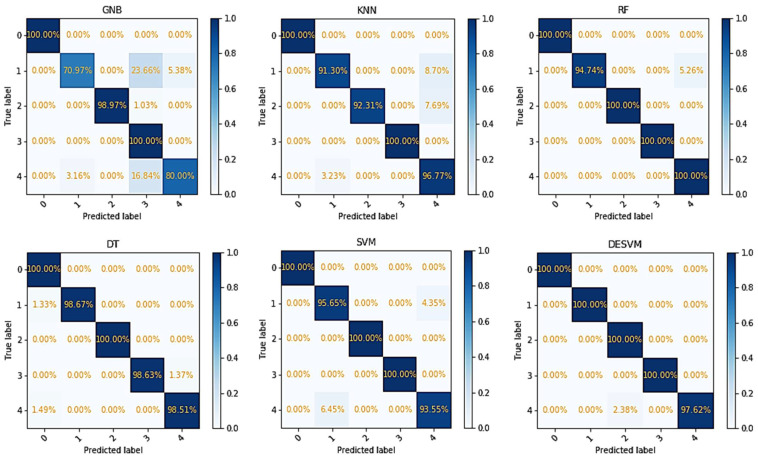
The confusion matrix of the recognition results for GNB, DT, KNN, RF, SVM, and DESVM machine learning algorithm.

The confusion matrix of the recognition results for each machine learning algorithm is shown in Figure 9. The vertical axis represents the truth, and the horizontal axis represents the output of the classification. The labels correspond to the test objects.

## 5. Conclusions

We have proposed an efficient object recognition algorithm (DESVM) based on purely tactile sensory data. The algorithm optimizes the parameters of the Gaussian kernel function of the support vector machine by taking advantage of the high search accuracy of the improved differential evolution algorithm. We increased the accuracy of tactile perception object recognition to 99.6%. The experimental results show that the method effectively improves the object recognition accuracy of tactile perception data. We provide an important reference for research in the field of tactile perception. In future studies, we will further increase the available datasets in the tactile field. In addition, we will further apply the proposed method to more tactile data with complex features to improve the generalizability of the method. 

## Figures and Tables

**Table 1:** Dataset division.

Objects	Cube Wood Block	Cuboid Plastic Block	Cylindrical Curved Box	Elliptical Surface	Irregular Surface Objects
Training set	70	70	70	70	70
Testing set	30	30	30	30	30
Total	Training set: 350, testing set: 150				

**Table 2:** Comprehensive performance of each machine learning model.

Algorithm	Accuracy	Precision	Recall	F1
GNB	0.8560	0.9148	0.8560	0.8411
KNN	0.9667	0.9687	0.9667	0.9669
DT	0.9733	0.9735	0.8670	0.8456
RF	0.9860	0.9832	0.9846	0.9859
SVM	0.9850	0.9821	0.9856	0.9822
DESVM	0.9960	0.9961	0.9960	0.9950

**Table 3:** The average (Avg) and standard deviation (Std) of the accuracy for ten-fold cross-validation.

Algorithm	Avg. Accuracy	Std. Accuracy
GNB	0.882	0.032
KNN	0.964	0.022
DT	0.978	0.025
RF	0.988	0.008
SVM	0.986	0.017
DESVM	0.996	0.006
